# Dominant‐negative variant in *SLC1A4* causes an autosomal dominant epilepsy syndrome

**DOI:** 10.1002/acn3.51786

**Published:** 2023-05-16

**Authors:** Jonai Pujol‐Giménez, Ghayda Mirzaa, Elizabeth E. Blue, Giuseppe Albano, Danny E. Miller, Aimee Allworth, James T. Bennett, Peter H. Byers, Sirisak Chanprasert, Jingheng Chen, Daniel Doherty, Andrew B. Folta, Madelyn A. Gillentine, Ian Glass, Anne Hing, Martha Horike‐Pyne, Kathleen A. Leppig, Azma Parhin, Jane Ranchalis, Wendy H. Raskind, Elisabeth A. Rosenthal, Ulrike Schwarze, Sam Sheppeard, Samuel Strohbehn, Virginia P. Sybert, Andrew Timms, Mark Wener, University of Washington Center for Mendelian Genomics (UW‐CMG), Undiagnosed Diseases Network (UDN), Michael J. Bamshad, Suzanne M. Leal, Deborah A. Nickerson, Peter Anderson, Tamara J. Bacus, Elizabeth E. Blue, Katherine Brower, Kati J. Buckingham, Jessica X. Chong, Diana Cornejo Sánchez, Colleen P. Davis, Chayna J. Davis, Christian D. Frazar, Katherine Gomeztagle‐Burgess, William W. Gordon, Martha Horike‐Pyne, Jameson R. Hurless, Gail P. Jarvik, Eric Johanson, J. Thomas Kolar, Colby T. Marvin, Sean McGee, Daniel J. McGoldrick, Betselote Mekonnen, Patrick M. Nielsen, Karynne Patterson, Aparna Radhakrishnan, Matthew A. Richardson, Gwendolin T. Roote, Erica L. Ryke, Isabelle Schrauwen, Kathryn M. Shively, Joshua D. Smith, Monica Tackett, Gao Wang, Jeffrey M. Weiss, Marsha M. Wheeler, Qian Yi, Xiaohong Zhang, Maria T. Acosta, Margaret Adam, David R. Adams, Raquel L. Alvarez, Justin Alvey, Laura Amendola, Ashley Andrews, Euan A. Ashley, Carlos A. Bacino, Guney Bademci, Ashok Balasubramanyam, Dustin Baldridge, Jim Bale, Michael Bamshad, Deborah Barbouth, Pinar Bayrak‐Toydemir, Anita Beck, Alan H. Beggs, Edward Behrens, Gill Bejerano, Hugo J. Bellen, Jimmy Bennett, Beverly Berg‐Rood, Jonathan A. Bernstein, Gerard T. Berry, Anna Bican, Stephanie Bivona, Elizabeth Blue, John Bohnsack, Devon Bonner, Lorenzo Botto, Brenna Boyd, Lauren C. Briere, Gabrielle Brown, Elizabeth A. Burke, Lindsay C. Burrage, Manish J. Butte, Peter Byers, William E. Byrd, John Carey, Olveen Carrasquillo, Thomas Cassini, Ta Chen Peter Chang, Sirisak Chanprasert, Hsiao‐Tuan Chao, Gary D. Clark, Terra R. Coakley, Laurel A. Cobban, Joy D. Cogan, Matthew Coggins, F. Sessions Cole, Heather A. Colley, Heidi Cope, Rosario Corona, William J. Craigen, Andrew B. Crouse, Michael Cunningham, Precilla D'Souza, Hongzheng Dai, Surendra Dasari, Joie Davis, Jyoti G. Dayal, Esteban C. Dell'Angelica, Katrina Dipple, Daniel Doherty, Naghmeh Dorrani, Argenia L. Doss, Emilie D. Douine, Dawn Earl, David J. Eckstein, Lisa T. Emrick, Christine M. Eng, Marni Falk, Elizabeth L. Fieg, Paul G. Fisher, Brent L. Fogel, Irman Forghani, William A. Gahl, Ian Glass, Bernadette Gochuico, Page C. Goddard, Rena A. Godfrey, Katie Golden‐Grant, Alana Grajewski, Don Hadley, Sihoun Hahn, Meghan C. Halley, Rizwan Hamid, Kelly Hassey, Nichole Hayes, Frances High, Anne Hing, Fuki M. Hisama, Ingrid A. Holm, Jason Hom, Martha Horike‐Pyne, Alden Huang, Sarah Hutchison, Wendy Introne, Rosario Isasi, Kosuke Izumi, Fariha Jamal, Gail P. Jarvik, Jeffrey Jarvik, Suman Jayadev, Orpa Jean‐Marie, Vaidehi Jobanputra, Lefkothea Karaviti, Shamika Ketkar, Dana Kiley, Gonench Kilich, Shilpa N. Kobren, Isaac S. Kohane, Jennefer N. Kohler, Susan Korrick, Mary Kozuira, Deborah Krakow, Donna M. Krasnewich, Elijah Kravets, Seema R. Lalani, Byron Lam, Christina Lam, Brendan C. Lanpher, Ian R. Lanza, Kimberly LeBlanc, Brendan H. Lee, Roy Levitt, Richard A. Lewis, Pengfei Liu, Xue Zhong Liu, Nicola Longo, Sandra K. Loo, Joseph Loscalzo, Richard L. Maas, Ellen F. Macnamara, Calum A. MacRae, Valerie V. Maduro, AudreyStephannie Maghiro, Rachel Mahoney, May Christine V. Malicdan, Laura A. Mamounas, Teri A. Manolio, Rong Mao, Kenneth Maravilla, Ronit Marom, Gabor Marth, Beth A. Martin, Martin G. Martin, Julian A. Martínez‐Agosto, Shruti Marwaha, Jacob McCauley, Allyn McConkie‐Rosell, Alexa T. McCray, Elisabeth McGee, Heather Mefford, J. Lawrence Merritt, Matthew Might, Ghayda Mirzaa, Eva Morava, Paolo Moretti, John Mulvihill, Mariko Nakano‐Okuno, Stanley F. Nelson, John H. Newman, Sarah K. Nicholas, Deborah Nickerson, Shirley Nieves‐Rodriguez, Donna Novacic, Devin Oglesbee, James P. Orengo, Laura Pace, Stephen Pak, J. Carl Pallais, Christina G.S. Palmer, Jeanette C. Papp, Neil H. Parker, John A. Phillips, Jennifer E. Posey, Lorraine Potocki, Barbara N. Pusey Swerdzewski, Aaron Quinlan, Deepak A. Rao, Anna Raper, Wendy Raskind, Genecee Renteria, Chloe M. Reuter, Lynette Rives, Amy K. Robertson, Lance H. Rodan, Jill A. Rosenfeld, Natalie Rosenwasser, Francis Rossignol, Maura Ruzhnikov, Ralph Sacco, Jacinda B. Sampson, Mario Saporta, Judy Schaechter, Timothy Schedl, Kelly Schoch, Daryl A. Scott, C. Ron Scott, Vandana Shashi, Jimann Shin, Edwin K. Silverman, Janet S. Sinsheimer, Kathy Sisco, Edward C. Smith, Kevin S. Smith, Lilianna Solnica‐Krezel, Ben Solomon, Rebecca C. Spillmann, Joan M. Stoler, Kathleen Sullivan, Jennifer A. Sullivan, Angela Sun, Shirley Sutton, David A. Sweetser, Virginia Sybert, Holly K. Tabor, Queenie K.‐G. Tan, Amelia L. M. Tan, Mustafa Tekin, Fred Telischi, Willa Thorson, Cynthia J. Tifft, Camilo Toro, Alyssa A. Tran, Rachel A. Ungar, Tiina K. Urv, Adeline Vanderver, Matt Velinder, Dave Viskochil, Tiphanie P. Vogel, Colleen E. Wahl, Melissa Walker, Stephanie Wallace, Nicole M. Walley, Jennifer Wambach, Jijun Wan, Lee‐kai Wang, Michael F. Wangler, Patricia A. Ward, Daniel Wegner, Monika W. Hubshman, Mark Wener, Tara Wenger, Monte Westerfield, Matthew T. Wheeler, Jordan Whitlock, Lynne A. Wolfe, Kim Worley, Changrui Xiao, Shinya Yamamoto, John Yang, Zhe Zhang, Stephan Zuchner, Michael J. Bamshad, Fuki M. Hisama, Gail P. Jarvik, Katrina M. Dipple, Matthias A. Hediger, Andrew B. Stergachis

**Affiliations:** ^1^ Department of Nephrology and Hypertension University Hospital Bern, Inselspital Bern Switzerland; ^2^ Department of Biomedical Research University of Bern Bern Switzerland; ^3^ Center for Integrative Brain Research Seattle Children's Research Institute Seattle Washington USA; ^4^ Department of Pediatrics University of Washington Seattle Washington USA; ^5^ Brotman Baty Institute for Precision Medicine Seattle Washington USA; ^6^ University of Washington, Institute of Public Health Genetics Seattle Washington USA; ^7^ Department of Laboratory Medicine and Pathology University of Washington School of Medicine Seattle Washington USA; ^8^ Department of Medicine University of Washington School of Medicine Seattle Washington USA; ^9^ Center for Developmental Biology and Regenerative Medicine Seattle Children's Research Institute Seattle Washington USA; ^10^ Department of Laboratories Seattle Children's Hospital Seattle Washington USA; ^11^ Group Health Cooperative Kaiser Permanente Washington Seattle Washington USA; ^12^ Genome Sciences University of Washington School of Medicine Seattle Washington USA

## Abstract

SLC1A4 is a trimeric neutral amino acid transporter essential for shuttling L‐serine from astrocytes into neurons. Individuals with biallelic variants in *SLC1A4* are known to have spastic tetraplegia, thin corpus callosum, and progressive microcephaly (SPATCCM) syndrome, but individuals with heterozygous variants are not thought to have disease. We identify an 8‐year‐old patient with global developmental delay, spasticity, epilepsy, and microcephaly who has a *de novo* heterozygous three amino acid duplication in *SLC1A4* (L86_M88dup). We demonstrate that L86_M88dup causes a dominant‐negative N‐glycosylation defect of SLC1A4, which in turn reduces the plasma membrane localization of SLC1A4 and the transport rate of SLC1A4 for L‐serine.

## Introduction

Serine synthesis is confined to glia within the brain and is shuttled to neurons via SLC1A4, a dedicated neutral amino acid transporter. Disruptions in this process lead to SPATCCM syndrome, which is characterized by seizures, microcephaly, spasticity, intellectual disability, developmental delay, and a thin corpus callosum with delayed myelination and cortical atrophy.^1^ Eight distinct variants within *SLC1A4* (Y191*, E256K, G374R, G381R, R457W, R457Q, L315Hfs*42, and W453*)^1^, ^2^, ^3^, ^4^, ^5^, ^6^, ^7^, ^8^ have been associated with SPATCCM syndrome in the recessive state. Notably, SLC1A4 haploinsufficiency does not appear to result in disease, as individuals heterozygous for the pathogenic Y191*, and L315Hfs*42 variants are unaffected. Furthermore, although SLC1A4 forms a trimeric protein, the *SLC1A4* R457W and E256K missense variants do not appear to impact protein folding or trafficking to the plasma membrane, but rather markedly reduce SLC1A4 L‐serine transport capacity.^8^ In contrast, we identified a patient with SPATCCM syndrome who was found to have a *de novo* heterozygous variant in *SLC1A4* with no rare coding or non‐coding *SLC1A4* variants *in trans*. As SLC1A4 forms a homomeric protein complex essential for shuttling L‐serine from astrocytes into neurons, we sought to evaluate whether this variant protein is exerting a dominant‐negative effect on the remaining wild‐type SLC1A4 protein. Dominant‐negative variants have been well characterized in similar transporters,^9^ and are characterized by their ability to co‐assemble with and subsequently interfere with the function of wild‐type protein via reduced overall plasma membrane expression or altered transport function.

## Methods

### Genetic testing of proband

Initial genetic testing included microarray (Seattle Children's Hospital, Seattle, WA, USA), chromosomal breakage studies from blood and fibroblasts (OHSU, Portland, OR), proband‐only exome sequencing (Baylor University, Waco, TX, USA), and trio exome sequencing (Prevention Genetics, Marshfield, WI, USA). Exome reanalysis and trio genome sequencing were performed through the Undiagnosed Diseases Network, and long‐read genome sequencing (Oxford Nanopore) was performed for the proband.

### Plasmid constructs, cell culture, and transfection

HEK293T cells (ATCC) were maintained under standard culture conditions^9^ and transfected using FuGENE HD (Promega, Madison, WI, USA) with a pcDNA 3.1 plasmid containing wild‐type human *SLC1A4* (NM_003038.5) tagged with a C‐terminal HA‐tag (*SLC1A4*_*wt*_) or a version of this plasmid with the sequence CTGCGCATG inserted at coding position 264 (*SLC1A4*_*L86_M88dup*_). Cells were transfected with 0.1 μg (Fig. 2) or 3 μg (Fig. 3) DNA/well, while co‐transfected cells received 0.05 μg (Fig. 2) or 1.5 μg (Fig. 3) DNA/well of each construct in a 1:1 ratio. Experiments were performed 24–48 h post‐transfection.

### Surface biotinylation and immunoblotting

Transfected cells were washed thrice then incubated for 1 h at 4°C with Biotinylation buffer (1.5 mg/mL Sulfo‐NHS‐SS‐Biotin, 10 mmol/L TEA pH 7.4, 2 mM CaCl_2_ and 150 mmol/L NaCl), followed by 20‐min at 4°C with quenching buffer (PBS‐Ca‐Mg supplemented with 100 mmol/L glycine) and then washed thrice. Cells were lysed using RIPA buffer (150 mmol/L NaCl_2_, 5 mmol/L EDTA, 1% Triton X‐100, 0.5% deoxycholate, 0.1% SDS, and 50 mmol/L Tris–HCl, pH 7.4, cOmplete™ PI). Cleared lysates were incubated with streptavidin‐agarose beads overnight at 4°C. Beads were centrifuged and the “non‐membrane” supernatant was collected. Beads were then washed thrice with solution A (with 50 mmol/L NaCl, 5 mmol/L EDTA, and 50 mmol/L Tris–HCl, pH 7.4), twice with solution B (500 mmol/L NaCl and 20 mmol/L Tris–HCl, pH 7.4), and once with solution C (10 mmol/L Tris–HCl, pH 7.4) and “membrane surface proteins” were eluted using Laemmli 2× buffer at 95°C. Samples were resolved on 8% SDS‐polyacrylamide (SDS‐PAGE) gels, transferred onto polyvinylidene difluoride membrane, immunoblotted using mouse‐monoclonal anti‐HA (Merck, South San Francisco, CA, USA) or anti‐Actin (Santa Cruz Biotechnology, Santa Cruz, CA, USA), incubated with HRP‐conjugated goat anti‐mouse IgG (Bio‐Rad, Hercules, CA, USA), and visualized with enhanced chemiluminescence (ECL). All biotinylated proteins were also visualized with Avidin–HRP conjugate (Bio‐Rad) to ensure equal sample loading. Optical densitometry was determined using ImageJ. To identify N‐glycosylated proteins, the non‐membrane fraction was treated with PNGaseF (NEB) prior to immunoblotting.

### Radiolabeled L‐Serine and L‐Alanine uptake

Transfected cells were washed thrice with Choline buffer (140 mmol/L choline chloride, 5 mmol/L KCl, 1 mmol/L KH_2_PO_4_, 1.8 mmol/L CaCl_2_, 0.4 mmol/L MgCl_2_, and 5 mmol/L HEPES pH 7.2) and then incubated for 15 min at room temperature (RT) with Choline buffer, and 4 min at RT with uptake solution (140 mmol/L NaCl, 5 mmol/L KCl, 1 mmol/L KH_2_PO_4_, 1.8 mmol/L CaCl_2_, 0.4 mmol/L MgCl_2_, and 5 mmol/L HEPES pH 7.2) supplemented with indicated amounts of non‐radioactive L‐Serine or L‐Alanine and 0.05 μCi of [^3^H]‐L‐Serine or [^3^H]‐L‐Alanine. Reactions were stopped by washing cells thrice with ice‐cold Choline buffer supplemented with 1 mmol/L L‐Serine or L‐Alanine and then incubated with MicroScint™‐20 (PerkinElmer, Waltham, MA, USA) for 1 h at RT. Radioactivity was measured by scintillation counting with the MicroBeta^2^ microplate counter (PerkinElmer), and counts per minute were transformed into influx rates (pmol/min).^9^ Kinetic parameters were obtained using the Hill equation.

### Statistics

Unpaired Student's *t* tests or Mann–Whitney *U* tests established whether differences between experimental groups were significant (*P* < 0.05).

### Standard protocol approvals

This study was approved by the National Institutes of Health (NIH) Institutional Review Board (IRB) (IRB #15HG0130), and written informed consent was obtained from all participants in the study.

## Results

### Clinical phenotype of autosomal dominant SLC1A4‐related disease

We present an 8‐½‐year‐old girl with intractable epilepsy, spasticity with axial hypotonia, severe congenital‐onset microcephaly, intracranial calcifications, multiple dysmorphic facial features, bilateral limb reduction defect, lipodystrophy, skeletal abnormalities, and intellectual disability (Table S1). She is the only child of non‐consanguineous parents and was born after a pregnancy complicated by IUGR. Brain MRI demonstrated microcephaly with simplified gyral pattern, intracranial calcifications, thin corpus callosum, diffusely poor myelination, bilateral linear periventricular calcifications, and severe asymmetric microphthalmia. Family history was negative for congenital anomalies or epilepsy. Trio exome sequencing revealed a *de novo* heterozygous c.256_264dup (p.Leu86_Met88dup) variant in *SLC1A4* that is absent from gnomAD v3.2.1. Long‐read genome sequencing of the proband did not reveal any rare variants *in trans* with *SLC1A4* Leu86_Met88dup. Exome reanalysis and trio genome sequencing identified no additional pathogenic variants to explain her complex phenotype (Table S2). She died at age 10 due to complications of her disease.

### SLC1A4_L86_M88dup_ has decreased substrate transport

**Figure 1:**
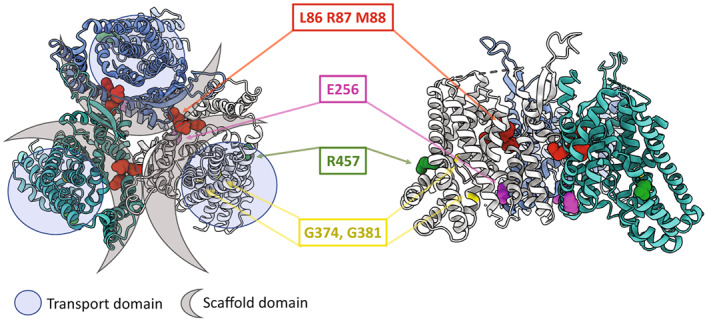
Structural relationship between pathogenic variants within SLC1A4. Upper and side view of the cryo‐EM structure of human SLC1A4. Transport and scaffold domains for each subunit of the trimer are highlighted as indicated. Previously reported disease‐linked *SLC1A4* variants (E256, R457, G374, and G381) are colored (pink, green, and yellow). Residues duplicated in the mutation under study L86, R87, and M88 are colored in red.

**Figure 2:**
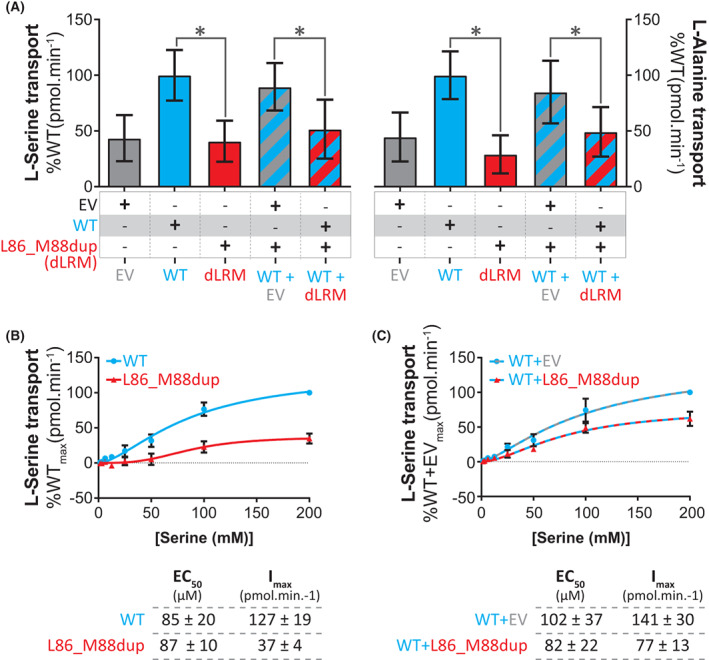
SLC1A4_L86_M88dup_ has a dominant‐negative impact on SLC1A4 function. (A) Uptake of 25 μmol/L [^3^H]‐L‐serine (left) and 25 μμmol/L [^3^H]‐L‐alanine (right) by HEK293T cells transfected with the indicated constructs. (B, C) [^3^H]‐L‐serine kinetics by HEK293T cells transfected with (B) SLC1A4_WT_ (WT) or SLC1A4_L86_M88dup_ (L86_M88dup), (C) or co‐transfected with both empty vector and SLC1A4_WT_ (EV + WT), or SLC1A4_WT_ and SLC1A4_L86_M88dup_ (WT + L86_M88dup). (below) Kinetic parameters obtained by fitting the results to the Hill equation. Data represented as mean ± SD and obtained from two independent experiments with 2–16 technical replicates each. *P < 0.001.

Residues L86‐R87‐M88 of SLC1A4 are located at the interface between the three SLC1A4 subunits and sit within the transmembrane helix 2 (TMH2) domain of SLC1A4.^10^ TMH2 is part of the scaffold domain of SLC1A4 (Fig. 1), which is essential for translocating substrates via an elevator transport mechanism.^11^, ^12^ We transfected HEK293T cells with either wild‐type SLC1A4 (SLC1A4_WT_) or Leu86_Met88dup variant SLC1A4 (SLC1A4_L86_M88dup_) to evaluate the transport ability of these proteins. Uptake of both L‐serine and L‐alanine in cells transfected with SLC1A4_L86_M88dup_ was significantly decreased (Fig. 2A), indicating that the Leu86_Met88dup variant impacts the overall transport activity of SLC1A4. Notably, whereas both SLC1A4_WT_ and SLC1A4_L86_M88dup_ have a similar affinity for L‐serine (Ec50 ≈ 80‐100 μmol/L), the maximum transport rate (*I*_max_) of SLC1A4_L86_M88dup_ was 3fold less than that of SLC1A4_WT_ (Fig. 2B).

### SLC1A4_L86_M88dup_ has a dominant‐negative impact on SLC1A4 function

As SPATCCM syndrome is associated with biallelic SLC1A4 loss of function variants, we examined whether SLC1A4_L86_M88dup_ disrupted SLC1A4_WT_ function in a dominant‐negative manner. We co‐transfected HEK293T cells with equal amounts of *both* SLC1A4_WT_ and SLC1A4_L86_M88dup_. Notably, these cells exhibited a significant loss of SLC1A4 transport function when compared to cells co‐transfected with equal amounts of SLC1A4_WT_ and empty vector, indicating that SLC1A4_L86_M88dup_ has a dominant‐negative impact on the transport activity of SLC1A4_WT_ for both L‐serine and L‐alanine (Fig. 2A). The dominant‐negative impact of SLC1A4_L86_M88dup_ on SLC1A4_WT_ function appears to be largely mediated by a decrease in the *I*_max_ of SLC1A4, as the affinity of SLC1A4 for L‐serine was not significantly changed (Fig. 2C).

### SLC1A4_L86_M88dup_ reduces the membrane localization of SLC1A4

**Figure 3:**
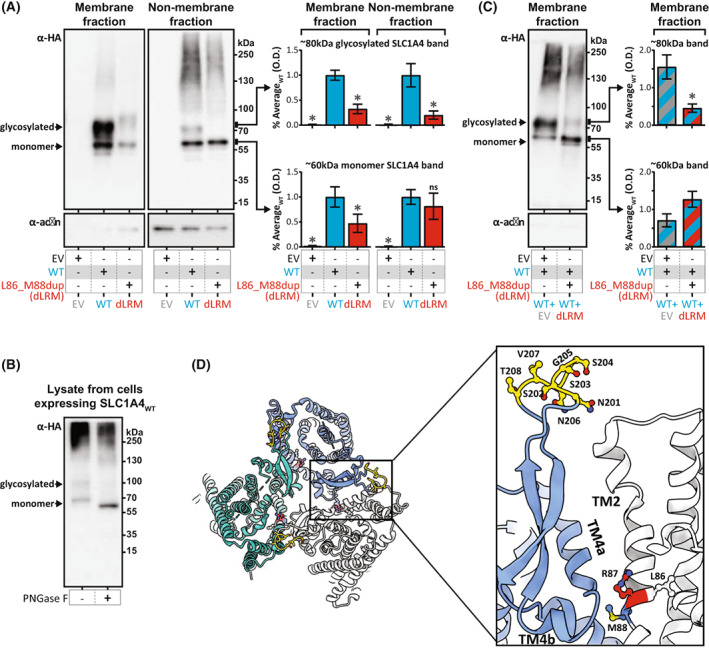
SLC1A4_L86_M88dup_ has a dominant‐negative impact on SLC1A4 N‐glycosylation. (A) Immunoblots showing the localization of overexpressed HA‐tagged SLC1A4_WT_ (WT) or SLC1A4_L86_M88dup_ (dLRM) within the plasma membrane and non‐membrane fractions. (below) Actin control. (right) Quantification of the optical density (O.D.) for the ~60 and ~80 kDa bands in the membrane and non‐membrane fractions (mean ± SD). ns, non‐significant *P* > 0.05; *P < 0.01. (B) Immunoblot showing the molecular weight of overexpressed HA‐tagged SLC1A4_WT_ (WT) in untreated cell lysates, as well as cell lysates treated with the N‐glycosidase PNGase F prior to the immunoblotting. (C) Same as (A), but using cells co‐transfected with both empty vector and SLC1A4_WT_ (EV + WT), or SLC1A4_WT_ and SLC1A4_L86_M88dup_ (WT + L86_M88dup). (D) Cryo‐EM structure of human SLC1A4 with residues Leu88, Arg87, and Met88 colored in red, blue, and white respectively. Putative N‐linked glycosylation sites^10^, ^16^ located in an extracellular loop present at the end of TM4 are colored in yellow.

We next sought to determine whether the impact of SLC1A4_L86_M88dup_ on SLC1A4 substrate transport was mediated by a reduction in SLC1A4 protein within the plasma membrane. Whereas the level of SLC1A4 monomers in the non‐membrane fraction was similar between cells transfected with either SLC1A4_L86_M88dup_ or SLC1A4_WT_, cells transfected with SLC1A4_L86_M88dup_ had a 2fold reduction in the amount of SLC1A4 in the membrane fraction (Fig. 3A). This is consistent with a model, whereby SLC1A4_L86_M88dup_ decreases substrate transport by restricting the amount of functional SLC1A4 protein within the plasma membrane, rather than affecting the transport cycle mechanism.

### SLC1A4_L86_M88dup_ impacts SLC1A4 N‐glycosylation

In the membrane fraction, we observed two predominant bands for SLC1A4, one corresponding to an SLC1A4 monomer (~60 kDa), as well as an additional band at ~80 kDa. As SLC1A4 is known to undergo N‐glycosylation,^13^ we treated these protein extracts with N‐Glycosidase PNGase F to determine whether this ~80 kDa band corresponds to N‐glycosylated SLC1A4. Indeed, the ~80 kDa band disappeared after treatment with PNGase F with a corresponding increased intensity of the monomeric ~60 kDa band (Fig. 3B), confirming the ~80 kDa band as N‐glycosylated SLC1A4. Notably, this N‐glycosylated 80 kDa band was largely absent in cells transfected with SLC1A4_L86_M88dup_ (Fig. 3A), indicating that SLC1A4_L86_M88dup_ disrupts normal N‐glycosylation of SLC1A4.

### SLC1A4_L86_M88dup_ has a dominant‐negative impact on SLC1A4 N‐glycosylation

We found that co‐transfection with both SLC1A4_WT_ and SLC1A4_L86_M88dup_ resulted in largely absent N‐glycosylated SLC1A4 (Fig. 3C), demonstrating a dominant‐negative impact of SLC1A4_L86_M88dup_ on SLC1A4_WT_ N‐glycosylation. Notably, the SLC1A4 TMH2 domain that contains residues L86‐R87‐M88 is not known to contain a glycosylation site, as glycosylation is thought to be limited to an extracellular loop present in the TM4 domain located over 100 amino acids C‐terminal to these residues.^10^ However, within the trimeric SLC1A4 structure, residues L86‐R87‐M88 directly interact with the TM4 domain of adjacent SLC1A4 monomers (Fig. 3D). Consequently, it is likely that the Leu86_Met88dup variant distorts the structure of the TMH2 and hinders the N‐glycosylation process of both the monomer containing the variant and the neighboring monomer within the trimeric protein.

## Discussion

We identify a patient with a clinical phenotype consistent with SPATCCM syndrome that appears to be caused by a heterozygous *de novo* variant in *SLC1A4*, thus implicating SPATCCM syndrome as both an autosomal recessive and dominant Mendelian disorder. Unlike *SLC1A4* variants associated with autosomal recessive SPATCCM syndrome, the *SLC1A4* Leu86_Met88dup variant has a dominant‐negative impact on SLC1A4, as is evidenced by its ability to interfere with the function of remaining wild‐type SLC1A4 protein. Specifically, SLC1A4_L86_M88dup_ results in an N‐glycosylation and membrane trafficking defect that reduces the overall membrane localization of wild‐type SLC1A4 and the transport rate of SLC1A4 for L‐serine. N‐glycosylation has been linked to trafficking of numerous transporters,^14^, ^15^ and the dominant‐negative impact of SLC1A4_L86_M88dup_ on N‐glycosylation indicates that N‐glycosylation likely occurs mainly after the formation of trimeric protein and is necessary for normal SLC1A4 trafficking and function. Overall, these findings provide basic insights into SLC1A4 trafficking and function and expand the spectrum of *SLC1A4*‐related disorders.

## Author Contributions

G.M., A.A., J.B., P.H.B., S.C., D.D., A.B.F., M.A.G., I.G., A.H., M.H‐P., K.A.L., A.P., W.R., S.Sh., S.St., V.P.S., A.T., M.W., M.J.B., K.M.D., F.M.H., G.P.J., and A.B.S., contributed to the evaluation of the patient's phenotype. G.M., E.E.B., S.S., J.C., E.A.R., U.S., A.B.S., J.R., and D.E.M. contributed to the analysis of genetic data. J.P‐G., A.B.S, E.E.B., and M.A.H. contributed to the *in vitro* study concept and design. J.P‐G., G.A., A.B.S., and M.A.H. contributed to the *in vitro* data acquisition and analysis. J.P‐G., A.B.S., E.E.B., G.M., and M.A.H. drafted the manuscript and figures.

## Conflict of Interest

DEM is on a scientific advisory board at Oxford Nanopore Technologies (ONT). DEM is engaged in a research agreement with ONT and they have paid for him to travel to speak on their behalf.

## Supporting information


**Table S1.** Clinical phenotype. This table provides the brain MRI findings and other phenotypic information about the proband described in this manuscript.

**Table S2.** Genetic testing results. This table provides information on the research and clinical genetic testing performed on the proband.
